# The Two-Faced Cytokine IL-6 in Host Defense and Diseases

**DOI:** 10.3390/ijms19113528

**Published:** 2018-11-09

**Authors:** Masashi Narazaki, Tadamitsu Kishimoto

**Affiliations:** 1 Department of Respiratory Medicine and Clinical Immunology, Graduate School of Medicine, Osaka University, Osaka 565-0871, Japan; 2 Department of Immunopathology, World Premier International Immunology Frontier Research Center, Osaka University, Osaka 565-0871, Japan; 3 Laboratory of Immune Regulation, World Premier International Immunology Frontier Research Center, Osaka University, Osaka 565-0871, Japan; kishimoto@ifrec.osaka-u.ac.jp

**Keywords:** cytokine, interleukin-6 (IL-6), IL-6 receptor, tocilizumab, inflammation, host defense, pathology

## Abstract

Interleukein-6 (IL-6), is produced locally from infectious or injured lesions and is delivered to the whole body via the blood stream, promptly activating the host defense system to perform diverse functions. However, excessive or sustained production of IL-6 is involved in various diseases. In diseases, the IL-6 inhibitory strategy begins with the development of the anti-IL-6 receptor antibody, tocilizumab (TCZ). This antibody has shown remarkable effects on Castleman disease, rheumatoid arthritis and juvenile idiopathic arthritis. In 2017, TCZ was proven to work effectively against giant cell arteritis, Takayasu arteritis and cytokine releasing syndrome, initiating a new era for the treatment of these diseases. In this study, the defensive functions of IL-6 and various pathological conditions are compared. Further, the diseases of which TCZ has been approved for treatment are summarized, the updated results of increasing off-label use of TCZ for various diseases are reviewed and the conditions for which IL-6 inhibition might have a beneficial role are discussed. Given the involvement of IL-6 in many pathologies, the diseases that can be improved by IL-6 inhibition will expand. However, the important role of IL-6 in host defense should always be kept in mind in clinical practice.

## 1. Introduction

Interleukein-6 (IL-6) is an essential cytokine that transmits defense signals from a pathogen invasion or tissue damage site to stimulate acute phase reactions, immune responses, hematopoiesis and various internal organs to prepare for host defense [1]. However, excessive and sustained production of IL-6 is associated with various inflammatory diseases [2,3,4,5]. A healthy serum IL-6 level is lower than 4 pg/mL but this level increases to several tens or even hundreds of pg/mL in chronic diseases depending on the disease severity and location. Values of more than 1000 pg/mL can occur during septic shock or a cytokine storm and in severe cases, a level measured in µg/mL can be reached, in which excessive defense signaling can threaten survival [6]. As IL-6 is involved in various diseases, it was proposed that its inhibition could improve the pathology of such conditions. The creation of an IL-6 inhibitory strategy started with the development of the anti-IL-6 receptor antibody, tocilizumab (TCZ). After observation of a drastic clinical effect of TCZ on Castleman disease, which is a typical inflammatory disease caused by IL-6 overproduction, TCZ was shown to have significant effects on rheumatoid arthritis (RA) and juvenile idiopathic arthritis (JIA). The safety profile of IL-6-targeting therapy has been confirmed through its use by a large number of patients with arthritis. Consequently, in 2017, TCZ was approved for treatment of giant cell arteritis, Takayasu arteritis and an artificial T cell therapy-induced cytokine releasing syndrome in leukemia/lymphoma. The successful results achieved with TCZ have changed the therapeutic strategies for these conditions and now, it is expected that IL-6-targeting therapy could be used in many refractory diseases; thus, clinical trials are underway.

## 2. Production of IL-6

Expression of IL-6 is regulated by various mechanisms that are gene polymorphism, chromatin remodeling, transcriptional and posttranscriptional levels [3]. A G/C polymorphism at −174 in the human *IL-6* flanking region affects the transcriptional level and is associated with inflammatory diseases [7,8]. The chromatin structure of the *IL-6* promoter is changed into accessible conformation for transcriptional factors during differentiation of immune cells such as monocyte [9]. Functional *cis*-regulatory elements in the human *IL-6* gene include nuclear factor kappa B (NF-κB) binding site (−74/−63 from the transcription start site), nuclear factor IL6 (NF-IL6) (also known as CCAAT/enhancer-binding protein beta or C/EBPβ) (−87/−76 and −159/−145), specificity protein 1 (SP1) (−109/−104 and −123/−119), cyclic AMP response element binding protein (CREB) (−165/−158), interferon regulatory factor 1 (IRF-1) (−267/−254) and activator protein 1 (AP-1) (−284/−277) [3]. Among these factors, NF-κB is a main transcriptional factor commonly activated by Toll-like receptors (TLRs)-mediated signals and pro-inflammatory cytokines including tumor necrosis factor α (TNFα), IL-1β and IL-17 [10,11].

**Figure 1:**
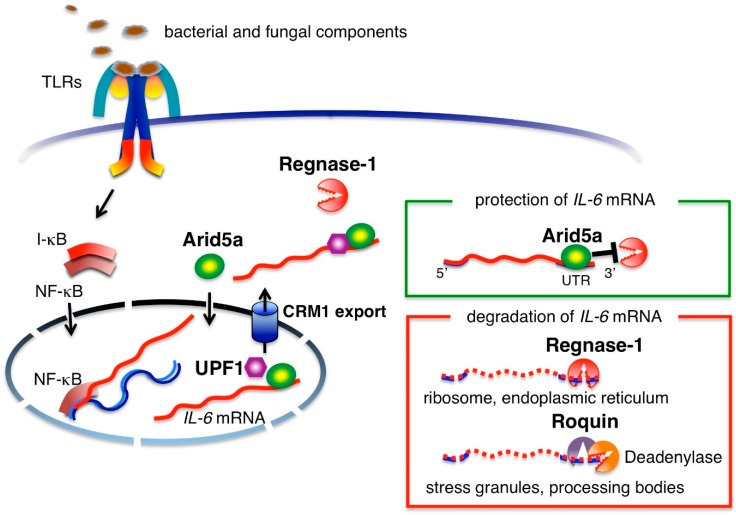
Transcriptional and post-transcriptional regulation of *interleukein-6* (*IL-6*) mRNA. Bacterial and fungal components and the molecules derived from tissue injury activate cell surface or intracellular Toll-like receptors (TLRs). Nuclear factor-kappa B (NF-κB) is a main transcriptional factor to induce transcription of *IL-6* mRNA. AT-rich interactive domain-containing protein 5a (Arid5a) translocates into the nucleus via an importin-α/β1 pathway and binds to the 3′UTR of *IL-6* mRNA. Then, Arid5a exports *IL-6* mRNA to the cytoplasm via the chromosomal region maintenance 1 (CRM1) pathway by binding to up-frameshift protein 1 (UPF1) and protects *IL-6* mRNA from degradation by Regulatory RNase-1 (Regnase-1). Regnase-1 degrades transcriptionally active *IL-6* mRNA by binding to the *IL-6* 3′UTR in the cytoplasm, endoplasmic reticulum and ribosomes. Another RNA-binding protein Roquin recognizes mRNAs overlapping with Regnase-1. However, Roquin degrades inactive mRNA by recruiting deadenylase in stress granules and processing bodies. The balance between the degradation of *IL-6* mRNA by Regnase-1 and the protection of *IL-6* mRNA by Arid5a controls the quantity of IL-6 production.

In infected lesions, bacterial and fungal components are recognized by cell surface TLRs and their nucleic acids are recognized by intracellular TLRs [12]. Noninfectious inflammation due to burn, trauma and even sterile surgical operations produces damaged, necrotic cells and a degraded extracellular matrix. The molecules derived from tissue injury are also recognized by TLRs [12,13]. Activation of cell surface and intracellular TLRs in monocytes and macrophages induces mRNA-transcription of *IL-6* and other pro-inflammatory cytokines, such as *TNFα* and *IL-1β*, via nuclear factor-kappa B (NF-κB) [10]. Signaling from TNFα or IL-1β also induces *IL-6* mRNA transcription [14]. After transcription, *IL-6* mRNA is regulated at the post-transcriptional level by adenylate-uridylate-rich elements (AREs) located in the 3′UTR region of *IL-6* mRNA (Figure 1). A nuclease known as regulatory RNase-1 (Regnase-1) degrades transcriptionally active *IL-6* mRNA by binding to *IL-6* 3′UTR in the cytoplasm, endoplasmic reticulum and ribosomes [15]. Macrophages from Regnase-1 knockout mice produce increased levels of IL-6 and the mice have been shown to spontaneously develop autoimmune diseases [16], indicating that post-transcriptional regulation of *IL-6* is critically linked to IL-6 production. Another RNA-binding protein, Roquin, degrades inactive mRNA in stress granules and processing bodies [15]. In contrast, RNA-binding protein, AT-rich interactive domain-containing protein 5a (Arid5a), is expressed in response to the presence of lipopolysaccharides (LPS), IL-1β and IL-6 in macrophages and under Th17 polarizing conditions in T cells. Arid5a is imported into the nucleus via an importin-α/β1 pathway and binds to the 3′UTR of *IL-6* mRNA. Then, Arid5a exports *IL-6* mRNA to the cytoplasm via the chromosomal region maintenance 1 (CRM1) pathway by binding to up-frameshift protein 1 (UPF1). The *IL-6* mRNA bound to Arid5a is protected from degradation by Regnase-1 in the cytoplasm [17,18]. The counteracting roles of Arid5a and Regnase-1 in regard to the stability of *IL-6* mRNA indicates that the balance between the two governs the quantity of IL-6 production [19].

## 3. IL-6 Receptor System and Signal Activation Modes

**Figure 2:**
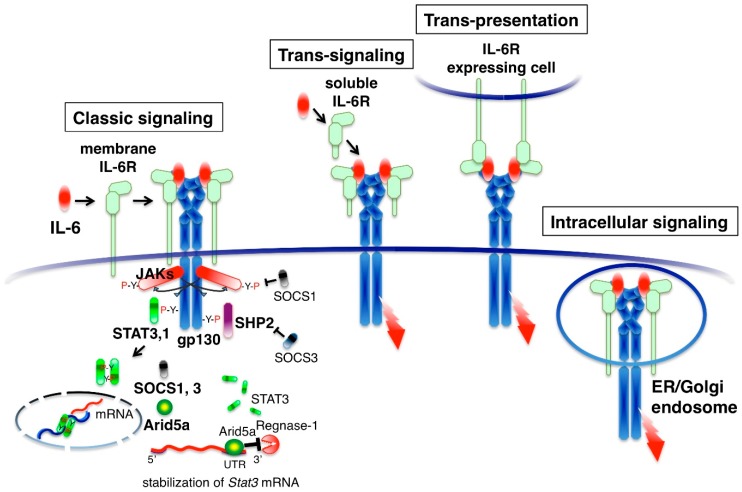
IL-6 signal transduction and modes of the IL-6 receptor system. IL-6 binds to the IL-6 receptor (IL-6R). The IL-6/IL-6R complex is associated with gp130, resulting in a hexamer structure. Homodimerization of gp130 activates Janus kinase (JAK) and subsequently activates Signal transducer and activator of transcription 3 (STAT3), STAT1 and SH2 domain-containing protein-tyrosine phosphatase 2 (SHP2). STAT3 and STAT1 move into the nucleus and SHP-2 activates the Ras-MAP kinase pathway. STAT3 induces the negative feedback molecules suppressor of cytokine signaling 1 (SOCS1) and SOCS3. SOCS1 inhibits JAKs. SOCS3 inhibits the SHP2 pathway. STAT3 also induces Arid5a, which stabilizes *Stat3* mRNA by binding to the *Stat3* 3′UTR. IL-6 signaling via membrane IL-6R or soluble IL-6R is called classic signaling or trans-signaling, respectively. When an IL-6R-expressing cell and a gp130-expressing cell are in close proximity, IL-6 is presented by between cells, a process called trans-presentation. IL-6 also activates gp130 in the endosomal compartment, endoplasmic reticulum (ER), or Golgi body through intracellular signaling. The red arrow indicates signal transduction.

IL-6 binds to its specific membrane receptor (IL-6R, also designated CD126) and the associated IL-6/IL-6R complex, inducing homodimerization of the signaling receptor component gp130 (also designated CD130). This results in the hexamer structure of the IL-6-signaling complex that consists of two molecules each of IL-6, IL-6R and gp130 (Figure 2). IL-6-intracellular signaling is initiated by the activation of cytoplasmic Janus kinases (JAKs: JAK1, JAK2 and TYK2) by homo-dimerization of gp130 and subsequent phosphorylation of tyrosine residues in the cytoplasmic region of gp130 [20,21]. Downstream signaling molecules, the signal transducer and activator of transcription 3 (STAT3), STAT1 and SH2 domain-containing protein-tyrosine phosphatase 2 (SHP2) are recruited to the tyrosine-phosphorylated motifs of gp130 [22]. STAT3 and STAT1 are then phosphorylated by the JAKs and translocated to the nucleus, generating the transcriptional output. SHP-2 activates the Ras-MAP kinase pathway [21]. The intracellular IL-6 signal is regulated by inhibitory or promoting molecules. STAT3 induces negative feedback molecules, which are suppressor of cytokine signaling 1 (SOCS1) and SOCS3 proteins [23,24]. SOCS1 binds to and inhibits JAK [25]. SOCS3 binds to gp130 and inhibits the SHP2 pathway [26]. STAT3 also induces the positive feedback molecule Arid5a, which selectively stabilizes *Stat3* mRNA by binding to *Stat3* 3′UTR and enhancing the STAT3 signal [27]. These negative or positive feedback mechanisms determine the magnitude of the IL-6 response.

Several modes of gp130 activation are known. IL-6 binds with high affinity to cells expressing both membrane IL-6R and gp130, such as hepatocytes, neutrophils, monocytes, activated B cells and CD4 T cells [2]. The activation of gp130 by membrane IL-6R is called classic signaling (Figure 2). Serum contains 60–100 ng/mL of soluble IL-6R, which is derived from the shedding of membrane IL-6R by the metalloproteases ADAM10 and ADAM17 [28]. IL-6 can also bind to the soluble form of IL-6R (sIL-6R) in serum. The IL-6/sIL-6R complex activates gp130-expressing cells, even if cells lack membrane IL-6R, a process known as trans-signaling [29]. Although cell types expressing membrane IL-6R are limited, gp130 is expressed in many cell types and it is thought that some aspects of the pleiotropic effect of IL-6 are derived from trans-signaling. Soluble gp130 (sgp130) is present in human serum and interacts with the IL-6/sIL-6R complex to inhibit trans-signaling [30]. When cells with membrane IL-6R and those with gp130 are in close proximity, IL-6 is presented by membrane IL-6R-expressing cells to the other gp130 expressing cells. This type of signaling is called trans-presentation [31,32]. IL-6 also activates gp130 in the endosomal compartment. Extracellular IL-6 is taken up by IL-6R and moves to the endosomal compartment by endocytosis where it activates gp130. Alternatively, newly synthesized IL-6 can transduce its signals in the endoplasmic reticulum or Golgi body before secretion [33]. When IL-6-producing cells express both IL-6R and gp130, autocrine stimulation may occur in cells as intracellular signaling [34].

The signaling component gp130 is shared with the cytokines of the IL-6 family, leukemia inhibitory factor (LIF), oncostatin M (OSM), ciliary neurotrophic factor (CNTF), cardiotrophin 1 (CT-1), IL-11, cardiotrophin-like cytokine factor 1 (CLCF1), IL-27 and IL-35 [35,36]. The concept that signal transducer gp130 is shared with IL-6 family cytokines explains the redundant actions of IL-6 family cytokines; however, the function of IL-6 is not always compensated by the other IL-6 family members. For example, some cytokines induce acute phase proteins in hepatocytes in vitro but acute phase proteins almost normalize when only IL-6 is suppressed in patients with inflammatory diseases. This suggests that the regulation of cytokine expression is different among IL-6 family members.

## 4. IL-6 in Host Defense and Disease

### 4.1. Acute Phase Protein Production, Regeneration and Immunity in the Liver

**Figure 3:**
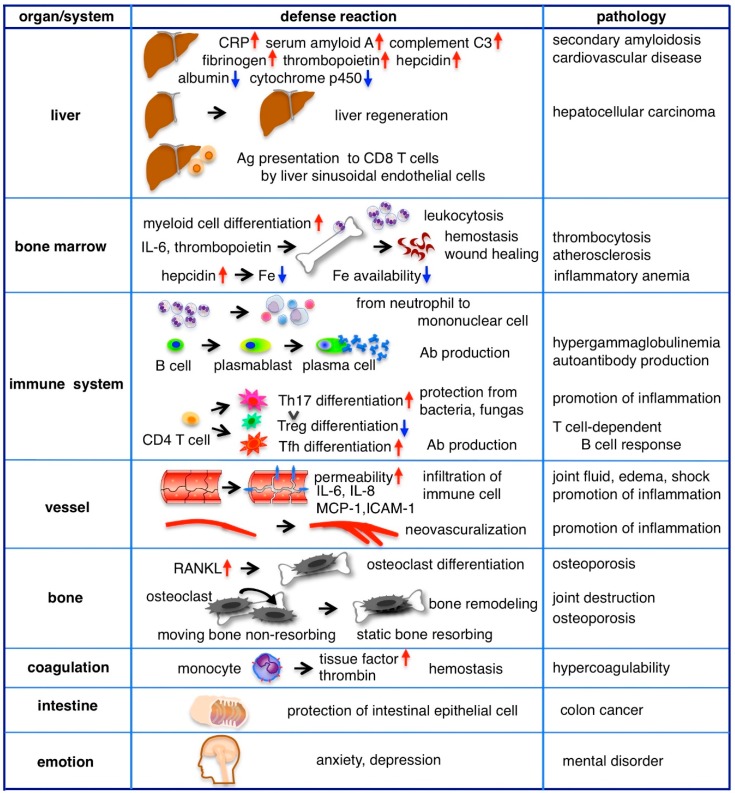
Defense reaction and pathological role of IL-6 in each organ. The defensive role of IL-6 in each organ is closely related to the pathological condition present. In the liver, IL-6 induces acute phase proteins, liver regeneration and CD8 T cell activation. Continuous elevation of IL-6 results in secondary amyloidosis, cardiovascular disease and hepatocellular carcinoma. In hematopoiesis, IL-6 induces leukocytosis by mobilization of neutrophils from the marginal pool into the circulation pool. IL-6 or IL-6-induced thrombopoietin induces thrombocytosis. IL-6-induced hepcidin reduces serum iron levels, leading to anemia. IL-6 plays a crucial role in neutrophil to mononuclear cell transition, B and T cell differentiation. High levels of IL-6 bound with sIL-6R induce vascular permeability in vessels and angiogenesis in chronic inflammatory sites. In bone homeostasis, IL-6 induces osteoclastogenesis. IL-6 activates the coagulation system for hemostasis, together with increases in fibrinogen and platelets, leading to an increased risk for the development of cardiovascular diseases. IL-6 also plays a protective role in intestinal epithelial cells. Accumulating evidence shows the association of IL-6 with a depressed mood. MCP-1: monocyte chemotactic protein 1, ICAM-1: intercellular adhesion molecule-1, RANKL: receptor activator of nuclear factor κB ligand.

The liver has long been considered the main target organ of IL-6 and in the past, IL-6 was alternatively called the hepatocyte stimulatory factor [37]. Since hepatocytes express membrane IL-6R and gp130, classic signaling mainly mediates IL-6 signaling in the liver. The liver quickly responds to IL-6 to help with host defense from the early phase of inflammation, producing various proteins, such as C-reactive protein (CRP), serum amyloid protein A, complement C3, haptoglobin, antitrypsin, fibrinogen and hematopoiesis regulatory proteins including hepcidin and thrombopoietin but reduces the production of albumin and cytochrome p450 (Figure 3). For example, CRP is a pattern recognition molecule that binds to specific shapes on the surfaces of pathogens and damaged cells. Ligand-bound CRP functions as an opsonin and can activate the classical complement pathway [38]. In clinical practice, the levels of acute phase proteins in serum are often measured and correlated with the degree of inflammation. In vitro, hepatocytes respond with the other IL-6 family cytokines to produce acute phase proteins; however, it has been shown that IL-6 deficient mice have a severely impaired acute phase response [39]. In addition, blocking the IL-6 receptor in patients with RA reduces acute phase protein levels [40], indicating that IL-6 is an essential cytokine in the induction of the liver’s acute phase response. The single nucleotide polymorphism rs2228145 leads to Asp358Ala substitution of IL-6R and is known to be associated with a reduction in membrane IL-6R, an increase in sIL-6R and reductions in CRP and fibrinogen, supporting the main role of membrane IL-6R-mediated classic signaling in acute phase protein induction [41]. Persistent high levels of serum amyloid A in chronic inflammation, such as RA or bronchiectasis, leads to secondary amyloidosis, which has various symptoms resulting from the deposition of amyloid A in tissues. Intractable diarrhea and malnutrition are caused by the deposition of amyloid A in the intestine. Urine protein and renal failure are caused by renal amyloidosis. Heart failure and arrhythmia are observed in cardiac amyloidosis.

In addition to the induction of acute phase proteins, IL-6 is also involved in liver regeneration and liver-mediated adaptive immunity [42]. The liver has the capacity to regenerate after injury or even hepatectomy. IL-6-deficient mice have been reported to show impaired liver regeneration that is characterized by liver necrosis and liver failure. However, IL-6 treatment rescues these mice by inducing hepatocyte proliferation and preventing liver damage [43]. IL-6 has anti-apoptotic effects on hepatocytes by inducing an anti-apoptotic member of the Bcl-2 family, Mcl-1, to promote hepatocyte growth [44]. However, despite its many positive effects, IL-6 is also associated with the development of hepatocellular carcinoma (HCC) after liver cirrhosis and elevated serum levels of IL-6 predict HCC progression in patients with chronic hepatitis B infection [45]. Thus, IL-6 is considered a serum marker of HCC and its over-expression has been associated with six-month mortality [46,47].

Antigen-stimulated antigen presenting cells (APCs) encounter naïve T cells in both the lymph nodes and spleen. In the peripheral tissues, the liver also stimulates naïve T cells to clear gut-derived pathogens [48]. Liver sinusoidal endothelial cells (LSECs) present antigens to naive CD8 T cells as APCs and mediate the IL-6 signal to CD8 T cells via trans-presentation using IL-6/IL-6R on the surface of LSECs [31]. Cytotoxic CD8 T cells, received with antigen presentation and IL-6 stimulation, rapidly express effector molecule granzyme B to induce apoptosis in target cells.

### 4.2. Hematopoiesis

Inflammation often accompanies with leukocytosis, thrombocytosis and anemia. IL-6 has an activity of myeloid blood cell differentiation. This activity was studied as macrophage and granulocyte inducer type 2A [49]. Administration of IL-6 to rabbits causes a biphasic pattern of neutrophilia [50]. At the first peak IL-6 mobilizes polymorphonuclear leukocytes (PMNs) from the marginated pool into the circulating pool at 2–6 h with a decrease of L-selectin expression of PMNs. At the second peak IL-6 releases PMNs from the postmitotic pool in the bone marrow at 12–24 h.

In vitro experiment indicates IL-6 acts a potent growth factor for megakaryocytes and promotes maturation of megakaryocytic colony formation together with IL-3 [51]. However, in inflammatory condition IL-6 indirectly controls platelet production by stimulating the liver to release Thrombopoietin (TPO). TPO is a major regulator of thrombopoiesis and is constitutively produced in hepatocytes [52]. Its concentration in plasma is regulated by binding to TPO receptor c-Mpl on the surfaces of platelets [53]. During inflammation, IL-6 induces TPO production in the liver and elevates its plasma level. IL-6-induced thrombocytosis is abrogated by the neutralization of TPO, suggesting that it is mediated by TPO [54]. Elevated levels of platelets contribute not only to primary hemostasis but also to host defense [55]. Platelets express TLR4 and present bound LPS to neutrophils, stimulating them to produce reactive oxygen species (ROS). Platelets also promote neutrophil extracellular traps (NETs) formation [56]. Activated platelets release many proteins that promote angiogenesis and wound healing, including platelet-derived growth factor-A (PDGF-A), B and C, insulin-like growth factor-1 and vascular endothelial growth factor (VEGF). On the other hand, chronic thrombocytosis and platelet activation are risk factors for atherosclerosis and related diseases, including myocardial infarction and stroke.

IL-6 indirectly controls erythropoiesis by stimulating the liver to release hepcidin [57]. Hepcidin is a master regulator of iron homeostasis, which binds to, internalizes and degrades the iron exporter ferroportin on duodenal enterocytes, macrophages and hepatocytes, resulting in hypoferremia. A reduction in plasma iron is thought to be a defense mechanism against extracellular infections by inhibiting iron availability to invading pathogens [58], whereas sustained elevation of hepcidin by chronic inflammation results in anemia due to the lack of iron availability for erythropoiesis. Anemia, together with high levels of fibrinogen and immunoglobulins, accelerates the erythrocyte sedimentation rate (ESR), which is correlated with the severity of inflammation and is often measured in clinical practice.

### 4.3. Immune Response

The cells responsible for immune reaction, such as neutrophils, monocytes, B cells and CD4 T cells, express membrane IL-6R and are stimulated by IL-6 classic signaling. Neutrophils accumulate early in the site of inflammation, followed by a more sustained population of mononuclear cells including monocytes, macrophages and lymphocytes. IL-6 orchestrates transition from neutrophils to mononuclear cells by enhancing neutrophil migration toward IL-8, which is produced by endothelial cells at the acute inflammation site [59,60]. Then, IL-8 reduces the adhesion molecule P-selectin glycoprotein ligand 1 (PSGL-1/CD162) on the surface of neutrophils, leading to an increase in their circulation [61]. IL-8 also stimulates neutrophils to release sIL-6R by proteolytical cleavage of IL-6R on the cell surface. Subsequently, IL-6/sIL-6R trans-signaling stimulates endothelial cells to secrete monocyte chemotactic protein-1 (MCP-1) and augment intercellular adhesion molecule-1 (ICAM-1) expression [62,63]. Through these processes, monocytes accumulate at inflammatory sites and firmly adhere to vascular endothelial cells.

IL-6 plays a pivotal role in shaping the acquired immune response by promoting the differentiation of B cells into immunoglobulin producing plasma cells and increasing the gamma globulin level in serum. IL-6 also supports the survival of plasmablasts which are precursors of plasma cells. Accordingly, hypergammaglobulinemia is typically observed in patients with Castleman disease, a condition involving the continuous production of IL-6 from affected lymph nodes. Elevated levels of gamma globulin are normalized by treatment with anti-IL-6R Ab, albeit usually not to levels below the lower limit of the normal range [64]. Further, IL-6 deficient mice have been reported to still produce antigen-specific Abs in vivo, despite the reduction of levels to less than half of those found in wild type mice [65]. Unlike acute phase protein synthesis, in which IL-6 plays an essential role, gamma globulin production does not appear to be completely dependent on IL-6.

In T cells, IL-6 regulates the direction of the specific differentiation of naïve CD4 T cells. IL-6 and transforming growth factor β (TGFβ) are coordinately required for Th17 differentiation. Th17 cells produce IL-17, IL-21 and IL-22 and contribute to protection against bacterial or fungal infection [66]. In these cytokines, IL-17 stimulates fibroblasts, epithelial cells and synoviocytes, leading to the secretion of a range of pro-inflammatory cytokines including IL-6, TNFα, IL-1β and various chemokines that play roles in neutrophil trafficking to the site of inflammation. IL-21 and TGFβ induce Th17 differentiation in a positive feedback loop to amplify Th17 cells. IL-22 induces the production of antimicrobial agents in keratinocytes for immune barrier function [67] and is crucial in host defense against Gram-negative bacterial infections in the lung [68]. IL-6 inhibits TGFβ-induced regulatory T cell (Treg) differentiation that plays a role in immune homeostasis. The resulting up-regulation of the Th17/Treg balance is thought to be involved in the development of various autoimmune disorders and chronic inflammation [69,70].

### 4.4. Blood Vessels

An increase in vascular permeability is a defense mechanism for immune cells to infiltrate tissues, remove pathogens and repair damaged tissues. This also allows immune-related proteins, such as cytokines, antibodies and complements, to be delivered to inflammatory sites. Vascular endothelial (VE)-cadherin is the main protein that mediates the adhesion of adjacent cells by homophilic binding, whereas its disassembly leads to vascular leakage. Endothelial cells do not express membrane IL-6R but do express gp130; thus, IL-6/sIL-6R trans-signaling stimulates endothelial cells to induce phosphorylation and internalization of VE-cadherin [71]. Inflammatory cytokines, including IL-6, induce the potent vascular permeability factor VEGF from adipose tissue or other cells and phosphorylate and internalize VE-cadherin in endothelial cells [72,73]. Swelling and fluid accumulation in the joints of patients with RA and peripheral pitting edema in RS3PE syndrome are explained by the enhanced vascular permeability caused by IL-6 or VEGF [74]. The large amount of IL-6 from affected lymph nodes induces a type of systemic edema, called anasarca, massive ascites fluid and pleural effusions that are observed in Castleman disease and TAFRO syndrome [75]. In addition, edema is enhanced by IL-6-mediated hypoalbuminemia in the liver. If very strong, acute and systemic inflammation occurs, excessive hyperpermeability leads to a shock vital state, as observed in sepsis or cytokine releasing syndrome. In this case, the level of IL-6 is correlated with prognosis. In the cecal ligation and puncture sepsis model, survival rates were improved by sgp130-Fc, which inhibits IL-6 trans-signaling [76], suggesting the importance of IL-6 trans-signaling inhibition in the systemic inflammatory response.

In order to supply oxygen and nutrients to the inflammatory lesion, new vessel formation from pre-existing vasculature to the inflammatory lesion is necessary [77]. IL-6 induces endothelial cell proliferation, migration and vascular vessel sprouting, with similar efficacy as VEGF [78]. New vessel formation in chronic inflammatory lesions of the RA joints is examined by power Doppler ultrasound assessment in clinical practice.

### 4.5. Bone Homeostasis

Bone fracture healing is a sequential process involving inflammation, regeneration and remodeling phases. Using the femur osteotomy model in mice, it was shown that the inhibition of IL-6 reduces inflammation, recruitment of immune cells and bone regeneration in the early phase and disturbs bone formation and remodeling in the repair phase, suggesting an essential role of IL-6 in bone healing [79]. On the other hand, chronic inflammation results in bone loss. Inflammatory cytokines, including IL-6, induce receptor activator of nuclear factor κB ligand (RANKL), an essential factor for osteoclastogenesis [80]. IL-6 transgenic mice show osteopenia—a decrease in osteoblasts and an increase in osteoclasts—resulting in severe alterations in cortical and trabecular bone microarchitecture [81]. Mature osteoclasts are classified into two different types based on their motility and function: static bone resorptive osteoclasts and moving non-resorptive osteoclasts. Intravital imaging has shown that anti-IL-6R Ab converts static bone resorbing osteoclasts to moving non-resorbing osteoclasts under inflammatory conditions [82]. The osteoclastogenesis-promoting actions of IL-6 have also been indicated in clinical situations where IL-6 inhibition has increased bone formation markers, decreased bone absorption markers and improved bone balance in patients with RA [40,83].

### 4.6. Coagulation System

IL-6 induces a membrane protein tissue factor (TF, also known as factor III or CD142) on the surfaces of monocytes [84]. TF promotes activation of the coagulation system by initiating the extrinsic coagulation pathway. It activates factors VIIa and Xa, following activating prothrombin to produce thrombin that cleaves fibrinogen to fibrin to generate fibrin clot formation. In an attempt to administer recombinant IL-6 to humans, IL-6 was shown to stimulate coagulation through an increase in thrombin-antithrombin III complexes and the prothrombin activation fragment F1 + 2 [85]. Conversely, activated coagulation factors induce IL-6. In a trial in healthy humans, the activation of coagulation through the administration of recombinant factor VIIa elicited an increase in plasma IL-6 [86]. The actions of IL-6, such as inflammation induction, fibrinogen concentration elevation, an increase in platelets and coagulation promotion, support the role of IL-6 in the development of cardiovascular disease [87,88].

### 4.7. Intestinal Tract

In addition to the role of IL-6 in the regeneration of the liver, IL-6 is involved in the protection of intestinal epithelial cells. In vitro, IL-6 induces anti-apoptotic proteins in colon epithelial cells against apoptosis. In a murine *Citrobactor rodentium* infection model, IL-6 deficiency resulted in marked apoptosis and ulcer formation in the colonic epithelium [89]. IL-6 deficiency has also been shown to induce severe colitis in a dextran sodium sulfate-induced colitis model [90]. These mice models suggest that IL-6 maintains intestinal barrier function and limits further inflammation. On the other hand, the effect of IL-6 on intestinal epithelial cells contributes to the promotion of colon cancer growth, invasion and metastasis [91,92].

### 4.8. Emotion and Behavior

There is a hypothesis that persistent depressive symptoms are based on host defense [93]. Following infection or injury, individuals react to dangerous situations with emotional change, which evokes behavioral alterations. A microarray analysis on whole blood from patients with major depressive disorder revealed that 165 genes were expressed differentially—the 90 overexpressed genes were associated with innate immunity and the 75 under-expressed genes were associated with the adaptive immune response [94]. A meta-analysis indicated that the inflammatory markers associated with depression are TNFα, IL-1, IL-6 and CRP [95] and there are many reports showing the association between serum IL-6 increase and depression [96]. The role of IL-6 in stress was also studied in a mouse model. The level of IL-6 increased with stress in wild-type mice; however, IL-6-deficient mice exhibited resistance to the development of depression-like behaviors by forced swim or tail suspension [97]. The behavioral effect of IL-6 was also demonstrated in mice subjected to continuously high levels of IL-6 through an IL-6-expressing virus. Levels of locomotor activity did not decline but novel object exploration and spontaneous alternation tests were disturbed in all mice with high levels of IL-6 [98].

## 5. Therapeutic Targeting of IL-6 in Diseases

### 5.1. Diseases in Which Tocilizumab (TCZ) Was Approved for Use in Japan, the EU or the US Before 2017

#### 5.1.1. Castleman Disease

**Figure 4:**
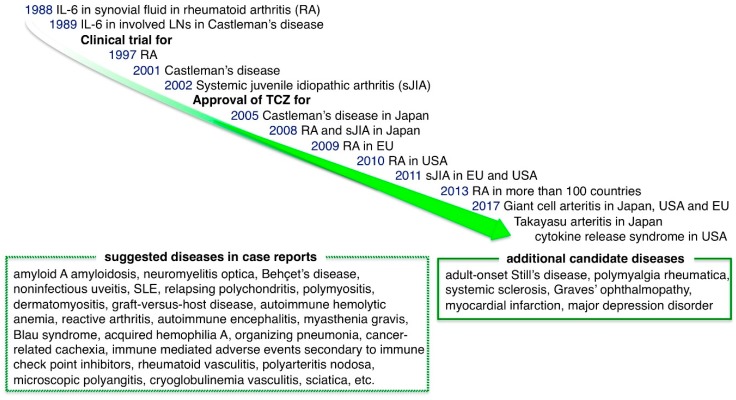
IL-6 inhibition therapy for various diseases. Currently, tocilizumab is approved for the treatment of Castleman disease, rheumatoid arthritis, systemic and polyarticular juvenile idiopathic arthritis, giant cell arteritis, Takayasu arteritis and cytokine releasing syndrome. IL-6 inhibition therapy is expected to constitute a novel therapeutic strategy for a wide range of diseases. Many case reports have also indicated that IL-6 inhibition is an effective treatment for various diseases. Clinical trials for some of them are ongoing.

The elucidation of the central role of IL-6 in inflammatory pathology led to the development of a groundbreaking drug that targets IL-6, tocilizumab (TCZ) (Figure 4) [36,99]. TCZ is a humanized anti-IL-6R IgG1 class monoclonal antibody that inhibits IL-6 binding to both membrane IL-6R and sIL-6R, blocking both IL-6 classic and trans-signaling. The first clinical success with TCZ was observed with Castleman disease, which was a good candidate for IL-6 inhibition because continuous production of IL-6 from affected lymph nodes is suggested to be responsible for the systemic inflammation that occurs in this disease [64,100]. A remarkable remission of inflammatory manifestations as well as improvements in anemia and the levels of CRP, fibrinogen and albumin were observed after the TCZ administration, with an accompanying disappearance of fever and fatigue. This provided the initial information on the effect of IL-6 inhibition on inflammatory conditions and TCZ was approved for Castleman disease in 2005 in Japan.

#### 5.1.2. Rheumatoid Arthritis (RA)

RA is a chronic inflammatory disease of the joints and surrounding tissues that is accompanied by joint pain, swelling, destruction and systemic inflammatory complications. Clinical trials of IL-6 inhibition therapy began due to the presence of high levels of IL-6 in synovial fluid in affected joints and the correlation between the serum IL-6 level and disease activity. After the promising results of the first randomized controlled trial of TCZ in RA [101], the efficacy of TCZ in RA was demonstrated by many clinical trials [4,102]. As with the observations in Castleman disease, laboratory parameters associated with RA were markedly improved after TCZ administration [40]. Some crucial studies showed the promising effect of TCZ compared to previous therapies. The AMBITION study, in which TCZ was compared with increasing doses of methotrexate (MTX), showed that TCZ performed better than MTX in terms of the response rate, remission rate and health-related quality of life [103]. In the ADACTA study, TCZ monotherapy improved disease activity scores to a greater extent than anti-TNFα adalimumab (ADA) monotherapy [104]. The U-Act-Early study and post-market surveillance indicated that immediately starting TCZ for patients with less advanced RA produces good results and leads to high rates of drug-free remission, suggesting the importance of stopping disease activity in the early phase [105]. TCZ was approved for RA treatment in 2008, 2009 and 2010 in Japan, the EU and the USA, respectively. The second anti-IL-6R Ab, sarilumab (SAR), is a fully human Ab that showed 10- to 40-fold higher affinity for IL-6R than TCZ. In a phase III study of RA patients, SAR with MTX produced significant improvements in symptoms and radiographic outcomes compared with placebo with MTX groups [106]. In the MONARCH study, SAR monotherapy resulted in greater improvement in disease activity scores than ADA [107]. SAR was approved for RA in 2017 in Japan, the EU and the USA.

#### 5.1.3. Systemic-Onset Juvenile Idiopathic Arthritis (sJIA)

sJIA, a type of chronic childhood arthritis with fever, skin rash, pericarditis, hepatosplenomegaly and growth retardation, was first described by George F. Still and is also known as Still’s disease. It is often treated with glucocorticoids but in the long-term, glucocorticoids cause a variety of serious side effects and growth impairment. IL-6 levels are correlated with the severity of disease manifestation, implicating IL-6 in the pathogenesis of sJIA [108]. A randomized phase III trial in children with refractory sJIA showed drastic improvement in disease activity by TCZ compared to conventional treatment [109]. Furthermore, body growth was restored and bone homeostasis improved in patients who received long-term treatment with TCZ [83,110]. Consequently, TCZ was approved for sJIA treatment in 2008 in Japan and in 2011 in the EU and the USA.

### 5.2. Diseases in Which TCZ Was Approved for Use in Japan, the EU, or the US in 2017

#### 5.2.1. Giant Cell Arteritis (GCA) and Takayasu Arteritis (TA)

The safety and effectiveness of TCZ for RA encouraged clinical trials of TCZ for various refractory inflammatory diseases. In 2017, the efficacy of TCZ was confirmed for diseases in a field other than inflammatory joint diseases. GCA is the most common large vessel vasculitis and cranial arteritis that affects individuals over the age of 50 and can lead to permanent visual loss. Most patients improve with glucocorticoids; however, some patients show disease flare after the reduction of treatment. A phase III GiACTA study involving 251 patients with GCA compared six months of treatment with prednisone taper plus TCZ (subcutaneous 162 mg) to prednisone taper alone. Fifty-six percent of patients receiving TCZ weekly and 53% receiving it every-other-week achieved sustained remission at 12 months compared to 14% in the six-month prednisone tapering alone group [111]. Treatment with TCZ plus prednisone led to a reduction in the cumulative dose of prednisone required to regulate GCA. In 2017, TCZ became the first GCA therapy to be approved by the USA, Japan and the EU. Another type of large vessel vasculitis is TA, often affecting women under the age of 40, in which arteries and their major branches narrow or cause aneurysms due to chronic inflammation of the vessel walls. Previously, glucocorticoids and immunosuppressive drugs, such as methotrexate or azathioprine, were usually used as treatment. In a phase III TAKT study, 19 patients with relapsed TA randomly received weekly TCZ (subcutaneous 162 mg) or placebo treatment. Although TCZ did not meet the defined primary endpoint for time to relapse TA, the hazard ratio for time to relapse was 0.41 (95.4% CI 0.15–1.10; *p* = 0.0596), suggesting a favorable effect of TCZ over the placebo [112]. A retrospective study of 46 patients with TA also showed the effectiveness of TCZ through significantly better event-free survival of patients who received TCZ compared with disease-modifying anti-rheumatic drugs (DMARDs) [113]. Japan approved TCZ for the treatment of TA in 2017. However, TA is known to frequently complicate inflammatory bowel disease [114]. Patients with inflammatory bowel disease should receive careful attention when receiving IL-6 inhibition therapy, because IL-6 has a protective effect of the intestinal epithelium.

#### 5.2.2. Cytokine Releasing Syndrome (CRS)

Treatment with TCZ is expected to be effective not only for chronic inflammatory diseases but also for acute inflammatory diseases. T cell-engaged therapy is a promising strategy for leukemia and lymphoma. In chimeric antigen receptor-modified T cell (CAR-T) therapy, for example, CD19-CAR-T is the engineered T cell that expresses an anti-CD19 single-chain variable fragment plus T cell receptor zeta and CD28 signaling domain. The CD19-CAR-T cell attacks B cell leukemia/lymphoma cells with B cell antigen CD19. Another method involves the CD19/CD3-bi-specific antibody (blinatumomab) that links T cells with CD19 cells. These therapies lead to a direct cytotoxic effect of T cells on target tumor cells. However, activated T cells produce massive amounts of IL-6, IL-8, IL-10, MCP-1 and IFNγ and induce an acute systemic inflammatory response, known as a cytokine storm or CRS. Severe cases of CRS are accompanied by life-threatening manifestations such as febrile neutropenia, hypotension, acute vascular leakage syndrome and respiratory distress syndrome. However, a case report showed that IL-6 inhibition with TCZ promptly reversed CRS [115]. A subsequent report showed that 18 of 39 patients with relapsed acute lymphoblastic leukemia who were treated with CAR-T developed grade 3–4 CRS but treatment with TCZ for 13 of them resulted in rapid improvement [116]. The drastic effect of TCZ on CRS opened up the possibility for the use of IL-6 blockade as a new strategy for an emergent fatal condition associated with acute cytokine storm. The USA approved TCZ for the treatment of CRS in 2017.

### 5.3. Diseases in Which the Efficacy of IL-6 Inhibition Was Demonstrated in Meta-Analysis or Phase II or III Clinical Trial.

#### 5.3.1. Adult-Onset Still’s Disease (AOSD)

The effectiveness of TCZ on some intractable diseases has been supported by a double-blind placebo-controlled randomized study and by numerous cases reporting its favorable effects. AOSD is an inflammatory syndrome involving fever, skin rash, hepatosplenomegaly and arthritis which, due to its similar symptoms, is thought to be the adult form of sJIA. It is characterized by elevated levels of inflammatory cytokines in serum including IL-6, IL-18, IL-1, TNFα and IFNγ. An increasing number of case reports or series has shown the effectiveness of TCZ on AOSD and reduction of glucocorticoid dose [117,118,119]. A meta-analysis of 10 studies with 147 patients showed that TCZ therapy achieved a partial remission rate in 85.38% and complete remission rate in 77.91%, and even in patients with refractory cases, the remission rate was 87.92% [120], suggesting a promising effect of TCZ on AOSD.

#### 5.3.2. Polymyalgia Rheumatica (PMR)

PMR is an inflammatory disease involving stiffness of the shoulders and pelvic girdles that occurs in people older than 50 years and is often associated with GCA. Usually, glucocorticoids are effective for treating PMR but some patients follow a chronic course and have glucocorticoid side effects. In a prospective study of 20 patients with active and recent onset PMR, all patients showed clinical improvement at 12 weeks after three TCZ (8 mg/kg) infusions at 4-week intervals [121]. A phase IIa trial involving newly diagnosed PMR patients also demonstrated the beneficial effects of TCZ on PMR [122]. All nine patients treated with intravenous TCZ (8 mg/kg) with a rapid tapering of glucocorticoids showed relapse-free remission without glucocorticoids after six months. Together, these reports suggest that TCZ is effective and reduces cumulative glucocorticoid doses in patients with PMR. Phase III clinical trial is being conducted (ClinicaTrials.gov NCT03263715).

#### 5.3.3. Systemic Sclerosis (SSc)

SSc is a refractory connective tissue disease characterized by tissue fibrosis that affects the skin, blood vessels, heart, lungs, kidneys and gastrointestinal tract. Currently, no adequate treatment has been developed to stop the progression of SSc; thus, therapeutic development is required [123]. The effects of IL-6 on keratinocyte proliferation promotion [124], collagen generation in dermal fibroblasts and differentiation from dermal fibroblasts into myofibroblasts may account for sclerotic changes in the skin of SSc patients [125]. The effect of IL-6 inhibition on sclerotic skin has been confirmed in bleomycin-induced scleroderma model mice [126]. Case reports of improvements in skin lesions and joint range of motion in SSc patients led to the conduction of a phase II study of 87 SSc patients [127,128]. The study showed that skin scores were not significantly improved following weekly subcutaneous treatment with TCZ but the percentage decrease in the predicted forced vital capacity at 48 weeks was significantly reduced compared to the control group [129]. Pulmonary symptoms associated with SSc are often refractory, thus confirmation of the validity of TCZ treatment is required. TCZ was reported to decrease skin scores in patients with short disease duration, elevated CRP and low IL-33 and CCL5, suggesting that certain types of SSc tend to respond to TCZ well [130]. A particular condition may have to be selected to obtain the effect of TCZ on the skin lesions of SSc. The results of a phase III trial (NCT02453256) are awaited.

#### 5.3.4. Graves’ Ophthalmopathy

There have been reports that TCZ may be an effective treatment for ophthalmic diseases. The clinical manifestations of Graves’ ophthalmopathy include upper eyelid retraction, edema and proptosis. Autoimmune inflammation of extraocular muscles and orbital fat is usually associated with hyperthyroidism [131]. Favorable effects of TCZ on Graves’ ophthalmopathy have been shown in some reports [132]. A randomized phase III clinical trial in 32 patients with glucocorticoid resistant Graves’ ophthalmopathy showed improved clinical activity scores and significant decreases in exophthalmos size following treatment with TCZ compared to a placebo [133]. A decrease in thyroid-stimulating immunoglobulin has also been reported in patients with thyroid-associated ophthalmopathy [134].

#### 5.3.5. Myocardial Infarction

IL-6 blockade therapy is also expected to work effectively against noninfectious local inflammation. IL-6 rapidly increases in response to ischemia in acute myocardial infarction [135]. In addition to CRP and amyloid A, IL-6 is associated with myocardial injury and mortality in acute coronary syndrome, which includes unstable angina and myocardial infarction [136]. This suggests that IL-6 contributes to the pathology of myocardial infarction. A double-blind randomized placebo-controlled phase II trial with 117 patients with non-ST-elevated myocardial infarction showed that a single 280 mg dose of TCZ significantly decreased the release of troponin T in patients who received TCZ within two days of symptom appearance and who were treated with coronary intervention [137]. The single dose of 280 mg used is much lower than that usually used for RA treatment, so further study is needed to clarify the effect of TCZ on myocardial infarction. A phase II clinical trial is being conducted to assess the effects of TCZ on myocardial infarction (NCT03004703).

#### 5.3.6. Depression

Many reports indicate a relationship between inflammatory cytokines and depression. In particular, TNFα and IL-6 have been associated with antidepressant-resistant depression [138]. Currently, it is thought that the next wave of antidepressants will target the immune system [139]. The first clinical trial of anti-cytokine Ab on depression was performed with TNFα inhibitors. A double-blind, placebo-controlled, randomized trial with anti-TNFα Ab infliximab in 60 patients with major depression showed an association between baseline levels of the inflammatory marker CRP and the response to infliximab [140]. Subsequently, a phase II double-blind placebo-controlled trial on depressive symptoms analyzed the effect of anti-IL-6 Ab sirukumab on RA and siltuximab on Castleman disease. Patients were grouped by the presence or absence of a prevalent depressed mood and anhedonia. An analysis of SF-36 questionnaire items on depressed mood and anhedonia indicated that significantly greater improvements in depressive symptoms were achieved with anti-IL-6 Abs compared with a placebo [141]. Another report, in which conventional synthetic DMARDs (csDMARDs) and biological DMARDs (bDMARDs) were indirectly compared using a network meta-analysis, demonstrated that bDMARDs performed better than csDMARDs for improving mental health. Among bDMARDs, IL-6 inhibition had a 90% probability of being the most effective for improving mental health outcomes [142]. Trials of IL-6 inhibition are being conducted in patients with depressive disorder using anti-IL-6 Ab sirukumab phase II (NCT02473289) and anti-IL-6R Ab TCZ phase II (NCT02660528).

### 5.4. Diseases in Which a Substantial Case Reports Showing TCZ Efficacy

#### 5.4.1. Amyloid A Amyloidosis

Clinical case reports describing the effectiveness of IL-6 inhibition on various diseases are accumulating. IL-6 is required for the production of the amyloid A protein in the liver; thus, IL-6 inhibition is thought to be effective for amyloid A amyloidosis. A typical case report showed that TCZ administration to patients with intestinal amyloidosis stopped diarrhea early and removed amyloid A deposition after three months of treatment [143]. Significantly superior effects of TCZ compared to those of anti-TNF agents were shown in a Japanese nationwide survey of 199 patients with amyloid A amyloidosis, which included RA (60.3%), uncharacterized inflammatory disorders (11.1%), neoplasms (7.0%) and other rheumatic diseases (6.5%) [144]. Case reports have supported TCZ treatment improved renal function and decreased frequency of fever attacks in patients with familial Mediterranean fever accompanied by amyloid A amyloidosis [145].

#### 5.4.2. Neuromyelitis Optica (NMO)

The efficacy of IL-6 inhibitory therapy has also been reported in intractable neurological diseases. NMO is a chronic demyelinating disease that is characterized by autoantibody against the astrocyte water channel protein aquaporin-4 (AQP-4). Autoantibodies induce the activation of complements, demyelination and necrosis in the optic nerve and spinal cord [146]. In vitro study showed that IL-6 enhanced the survival of plasmablasts and their anti-AQP-4 Ab secretion, whereas TCZ inhibited the survival of plasmablasts and the production of anti-AQP-4 Ab [147]. A pilot study of TCZ in seven refractory patients with NMO showed a marked decrease in the relapse rate and intractable pain [148], leading to a subsequent clinical phase II trials (NCT03350633, NCT03062579) and phase III trials (NCT02073279, NCT02028884).

#### 5.4.3. Behçet’s Disease

Behçet’s disease is characterized by the symptoms of oral aphtha, genital ulcers, skin lesions and uveitis. A glucocorticoid and immunosuppressant sparing effect of TCZ was reported for seven patients with refractory vasculo-Behçet’s disease [149]. In addition, case reports have suggested that TCZ has favorable effects on neuro-Behçet’s disease characterized by elevated levels of IL-6 in cerebrospinal fluid [150,151]. There have been several case reports in which TCZ showed effectiveness on refractory uveitis induced by Behçet’s disease [152], noninfectious cases [153], or JIA [154,155]. A phase II trial in Behçet’s uveitis is currently underway (NCT03554161).

#### 5.4.4. Systemic Lupus Erythematosus (SLE)

SLE is a typical autoimmune disease involving the activation of T and B cells that, despite few previous reports, is expected to be affected by IL-6 inhibition. In an open trial of 16 patients with SLE, TCZ improved the disease activity score in eight of the 15 evaluable patients. Arthritis symptoms improved in all seven patients with the condition. Anti-dsDNA Ab levels decreased by 47% in patients treated with 4 or 8 mg/kg of TCZ, whereas a 7.8% decrease in IgG levels was observed, suggesting the suppressive effect of TCZ on autoantibody-producing cells [156]. An analysis of circulating T and B cell subsets in patients with SLE showed that TCZ decreased the frequency of plasmablasts/plasma cells and memory B cells but increased the concentrations of antigen-inexperienced B cells and naïve T cells, suggesting the restoration of B and T cell homeostasis [157]. A case report also indicated that TCZ was effective for recurrent pleural and pericardial effusion in patients with SLE [158]. Further clinical studies are required to confirm the efficacy of TCZ on SLE.

#### 5.4.5. Relapsing Polychondritis (RP)

RP is an orphan disease involving refractory inflammation in cartilage with no standardized treatment. Responses of RP patients to TCZ treatment have varied; in one case report, a sustained response was shown by two patients [159] and others have indicated its effectiveness [160,161,162,163] but one report showed it to be ineffective [164]. A French multicenter retrospective cohort study with 41 patients with RP who were exposed to 105 biologics reported the efficacy of TCZ (n = 17) as well as TNFα inhibitors (n = 60) but a low number of complete responses and concerns about the risk of infection were reported [165].

#### 5.4.6. Inflammatory Myopathy

Inflammatory myopathies are characterized by autoantibody or immune cell-mediated responses. Only a limited number of cases have reported the effectiveness of TCZ for these conditions, including polymyositis [166], dermatomyositis [167] and anti-synthetase syndrome [168]. Among these diseases, a phase II clinical trial for polymyositis and dermatomyositis is underway (NCT02043548).

#### 5.4.7. Graft-Versus-Host Disease (GVHD)

GVHD after hematopoietic cell transplantation sometimes shows refractory manifestations. A retrospective evaluation involving the administration of TCZ (8 mg/kg) every two weeks in 16 patients with severe steroid-refractory acute GVHD of the lower gastrointestinal tract reported that ten patients achieved complete response after a median time of 11 days (range 2 to 28 days) [169]. A phase II trial in acute GVHD is currently being conducted (NCT01475162). GVHD with acquired hemophilia A is characterized by the presence of autoantibodies against coagulation factor VIII after hematopoietic cell transplantation. A case report showed that TCZ treatment was effective for GVHD with acquired hemophilia A [170].

### 5.5. Diseases in Which a Few Case Reports Showing TCZ Efficacy

Case reports have shown TCZ to be effective for a variety of other diseases, including autoimmune hemolytic anemia [171,172], reactive arthritis [173], autoimmune encephalitis [174,175], myasthenia gravis [176], Blau syndrome [177], anti-Caspre2 syndrome [178], refractory organizing pneumonia associated with Sjögren’s syndrome [179], cancer-related cachexia [180] and steroid refractory immune-mediated adverse events secondary to immune check point inhibitors [181]. Besides large vessel vasculitis, several case reports have indicated that TCZ has effects on rheumatoid vasculitis [182], polyarteritis nodosa [183,184], microscopic polyangiitis [185,186] and cryoglobulinemia vasculitis [187]. A report showed that epidural administration of TCZ reduced pain due to sciatica with lumbar spinal stenosis [188]. Although these case reports showed favorable results for TCZ, the effects have not yet been confirmed by randomized clinical trials. Further clinical studies are required to fully support the use of IL-6 inhibition in the daily clinical practice. Among patients with low frequency diseases, the number of patients who are refractory to conventional therapy is even smaller, sometimes complicating the obtainment of statistical significance in clinical trials, so organized multi-center trials are needed.

## 6. Safety of TCZ

Given the various roles of IL-6 in the immune system, concerns had been raised about a possible increase in the rate of serious infection associated with an IL-6 blocking strategy. Risk assessment of IL-6 inhibition has mainly been based on experience of TCZ treatment for RA patients. The safety information of TCZ was first reported from Japan [189]. The meta-analysis with 2188 patient-years (PYs) exposure reported the rate of serious infections was 6.22/100PYs. Abnormalities in the laboratory test such as increase in lipid parameters and liver enzymes but most were mild. The ACT-SURE study reported 5.1 serious infections per 100 PYs following TCZ treatment in 1680 patients with RA, which is similar to the rate previously observed for the anti-TNFα antibody adalimumab [190]. The multicenter retrospective cohort in Japan showed comparative risk of hospitalized infection among biologics infliximab (hazard ratio was 1.54, 95% confidence interval (CI): 0.78–3.04), adalimumab (1.72, 95% CI: 0.88–3.34), abatacept (1.11, 95% CI: 0.55–2.21) and tocilizumab (1.02, 95% CI: 0.55–1.87) compared with etanercept in the study with 1596 RA patients. However, RA functional class 3/4 (1.92, 1.20–3.09), body mass index < 18.5 (2.55, 95% CI: 1.57–4.14), prednisolone more than 7.5 mg/day (3.56, 95% CI: 2.15–5.88), prednisolone from 5 mg to 7.5 mg/day (1.88, 95% CI: 1.10–3.22) and chronic lung disease (1.85, 95% CI: 1.17–2.92) contributed more to the risk of hospitalized infection than biologics [191].

Decrease of neutrophil counts have been reported in trial of TCZ in RA patients, however TCZ-induced neutropenia does not appear to be associated with serious infection [192]. It is thought that the decrease of neutrophil by TCZ is not due to myelotoxic effect but due to the increase of margination of circulating neutrophils into the bone marrow [50]. Considering the protective effect of IL-6 on epithelial cells, concerns have been expressed about complications in the intestinal mucosa due to IL-6 inhibition. In the German Biologics Register, IL-6 inhibition by TCZ led to an increase in the incidence of lower intestinal perforation with a hazard ratio of 4.48 (95% CI: 2.0–10.0) in RA patients [193]. Patients with a past history of diverticulitis need to be careful with IL-6 inhibition therapy.

The largest surveillance study of TCZ was performed for 28 weeks in 7901 patients in Japan and reported a rate of serious adverse events of 9.6%, with the most common event being infection (3.8%) [194]. However, the standardized mortality ratio 1.15 (95% CI: 0.83–1.61) did not exceed the ratio between 1.46 (95% CI: 1.32–1.60) and 1.90 (95% CI: 1.75–2.07) previously reported in a Japanese RA cohort. Further, long-term observations of 4527 patients over three years indicated no increases in the proportions of patients with fatal events, serious infection, or malignancy [195].

## 7. Diseases Where IL-6 Inhibition Is Predicted to Be Effective

Considering the reported effective cases of TCZ to date, the diseases for which IL-6 inhibition might have beneficial role can be predicted. First, IL-6 inhibition is expected to affect diseases that can be pathologically related to long-term IL-6 action, such as Castleman disease and amyloid A amyloidosis. Secondly, diseases for which the IL-6 level is correlated with disease activity may be ameliorated by IL-6 inhibition, as evidenced by Castleman disease, RA, sJIA and large vessel vasculitis. However, IL-6 is involved in liver regeneration and intestinal epithelium protection, so it is possible that elevated levels of IL-6 protect organs from damage or promote recovery from dysfunction. For example, a pilot study involving TCZ treatment in Crohn’s disease is of interest, as a report stated that TCZ caused different outcomes in systemic inflammation and intestinal lesions. The acute phase inflammatory response of Crohn’s disease was completely suppressed by TCZ but endoscopic and histological examinations revealed no changes [196]. The beneficial role of IL-6 inhibition is not always certain, even if the IL-6 level is correlated with disease activity, so careful selection is necessary to decide which disease should be treated. Third, IL-6 inhibition may be useful for diseases caused by autoantibodies. Some case reports of TCZ in NMO and SLE have shown decreases in autoantibody levels, such as anti-AQP-4 Ab or anti-dsDNA Ab. A decrease in autoantibody levels over a reduction in total IgG levels suggests that IL-6 is deeply involved in autoantibody production. Several case reports have shown that TCZ acts effectively on autoantibody-mediated diseases, such as autoimmune hemolytic anemia, myasthenia gravis, acquired hemophilia A and anti-Caspre2 syndrome. Fourth, IL-6 inhibition may be effective for the prevention of cardiovascular disease, since IL-6 elevates platelet count, fibrinogen concentration and activity of coagulation. Atherothrombosis is initiated and progressed by inflammation [197]. Evidence has also shown that high levels of IL-6 are associated with cardiovascular disease risk [87,88]. A meta-analysis showed that the risk of coronary heart disease is reduced by 3.4% for every copy of IL-6R with Asp358Ala inherited, suggesting a relationship between IL-6R and coronary heart disease [198]. Fifth, IL-6 inhibition therapy may be an effective treatment for certain cancers in which IL-6 promotes proliferation [199]. When the growth of cancer depends on IL-6 autocrine signaling, the IL-6 signal may be mediated by a mode of intracellular signaling and inhibitors of IL-6 signal transduction such as JAK inhibitors are possibly effective [34].

## 8. Conclusions

IL-6 inhibitory therapy is applied worldwide as treatment for RA and JIA and is now being used for the treatment of large vessel vasculitis. IL-6 inhibitory therapy is expected to be used for other inflammatory diseases given its strong inflammation suppressing effect and safety profile. Various biologics that interact with the extracellular domain of the IL-6 receptor complex components and low molecular weight compounds that interact with intracellular signaling molecules are being developed one after the other [200]. A new type of IL-6 inhibitor, the sgp130-Fc fusion protein, that selectively inhibits trans-signaling has also been developed [76]. Options for inhibiting IL-6 action will further increase in the future. The growing effectiveness of IL-6 inhibition therapy against intractable diseases is a reminder of the history of glucocorticoids. The inflammatory suppressive effect of glucocorticoids was revealed when they were administered to patients with RA. Since then, glucocorticoids have been used for a large number of noninfectious inflammatory diseases; however, long-term use of glucocorticoids is accompanied by various side effects and careful attention should be paid in clinical practice. Given the involvement of IL-6 in many pathologies, the diseases that can be improved and treated by IL-6 inhibition therapy will expand. However, the important role of IL-6 in host defense should always be kept in mind.
